# Playing the Catch-Up Game: Accelerating the Scale-Up of Prevention of Mother-To-Child Transmission of HIV (PMTCT) Services to Eliminate New Pediatric HIV Infection in Nigeria

**DOI:** 10.1371/journal.pone.0169342

**Published:** 2017-01-03

**Authors:** Edward Adekola Oladele, Hadiza Khamofu, Seun Asala, Mariya Saleh, Uche Ralph-Opara, Charles Nwosisi, Chukwuma Anyaike, Catherine Gana, Oluwasanmi Adedokun, Rebecca Dirks, Olufunsho Adebayo, Modupe Oduwole, Justin Mandala, Kwasi Torpey

**Affiliations:** 1 FHI360, Abuja, Nigeria; 2 National AIDS and STI Control Programme, Federal Ministry of Health, Abuja, Nigeria; 3 FHI 360, Washington DC, United States of America; 4 UNAIDS Country Office, Abuja, Nigeria; 5 College of Health Sciences, University of Ghana, Accra, Ghana; University of North Carolina at Chapel Hill School of Dentistry, UNITED STATES

## Abstract

**Introduction:**

As the world is making progress towards elimination of mother-to-child transmission of HIV, poor coverage of PMTCT services in Nigeria remains a major challenge. In order to address this, scale-up was planned with activities organized into 3 phases. This paper describes the process undertaken in eight high burden Nigerian states to rapidly close PMTCT coverage gaps at facility and population levels between February 2013 and March 2014.

**Methods:**

Activities were grouped into three phases–pre-assessment phase (engagement of a wide range of stakeholders), assessment (rapid health facility assessment, a cross sectional survey using mixed methods conducted in the various states between Feb and May 2013 and impact modelling), and post-assessment (drawing up costed state operational plans to achieve eMTCT by 2015, data-driven smart scale-up).

**Results:**

Over a period of 10 months starting June 2013, 2044 facilities were supported to begin provision of PMTCT services. This increased facility coverage from 8% to 50%. A 246% increase was also recorded in the number of pregnant women and their families who have access to HIV testing and counselling in the context of PMTCT. Similarly, access to antiretrovirals for PMTCT has witnessed a 152% increase in these eight states between October 2013 and October 2014.

**Conclusion:**

A data-driven and participatory approach can be used to rapidly scale-up PMTCT services at community and facility levels in this region. These results present us with hope for real progress in Nigeria. We are confident that the efforts described here will contribute significantly to eliminating new pediatric HIV infection in Nigeria.

## Introduction

The global community is optimistic that an end to the AIDS epidemic is possible. The UNAIDS Gap report of 2014 boldly admitted that “we are at the beginning of the end of AIDS”. [1] Global data supports this optimism and shows progress in reduction of new infections among adults and children. In 2013, 240,000 [210,000–280,000] children were newly infected with human immunodeficiency virus (HIV). This is 58% lower than in 2002, the year with the highest number, when 580,000 [540,000–640,000] children became newly infected with HIV. Although slower than other regions, sub-Saharan Africa which is the region hit worst by the HIV epidemic, is also showing some progress. [1]

The rate of progress however is not uniform across different countries in sub-Saharan Africa. There are prevalent demand (uptake of services) and supply (low coverage and availability) issues that continue to account for the slow progress in this region. [2] Nigeria is one of the few countries with low coverage of prevention of mother-to-child transmission of HIV (PMTCT) services and corresponding little improvement in reduction of new paediatric infections. [3, 4] The number of new HIV infections among children in Nigeria has declined by only 19% since 2009 compared to 50% or more in other African countries such as Botswana, Ethiopia, Ghana, Malawi, Mozambique, Namibia, South Africa and Zimbabwe [1].

Nigeria accounts for more than 30% of the global PMTCT gap and continues to contribute the second highest proportion to the number of persons living with HIV globally. In addition, it is the country with the highest contribution to new paediatric infections in 2013. The 51,000 new HIV infections among children in Nigeria in 2013 accounted for one quarter of all new HIV infections among children in the 21 Global Plan priority countries. [1, 5] Coupled with other issues [6–8], limited access to PMTCT services, due to geographic, social and financial barriers, is a major challenge in Nigeria. As of 2012, only 1,320 (mostly public) of the over 26,000 facilities in the country offered PMTCT services [9, 10] reaching about 19% of pregnant mothers with HIV testing & counselling (HTC) and about 17% of HIV-positive mothers with antiretrovirals (ARVs) for PMTCT. [11]

The Global Plan to eliminate paediatric HIV infections set a PMTCT population coverage target of 90%. [5] Nigeria was not on course to meet these targets by 2015. By 2013, it was evident that key innovative strategies had to be employed to rapidly close coverage gaps and increase access to PMTCT services.

In realization that aggregate level data masks sub-national differences, the Government of Nigeria identified 12 of its 36 states and the Federal Capital Territory which account for 70% of the country’s MTCT burden. These were then tagged the priority 12+1 states for PMTCT expansion. FHI 360 provides support to eight of these 12+1 states for HIV/AIDS service provision. These eight states are: Abia, Akwa Ibom, Anambra, Bayelsa, Cross River, Kano, Lagos and Rivers states.

In this paper, we describe the process undertaken in these eight high burden states to close PMTCT coverage gaps at the facility and population levels.

## Methods

The intervention was grouped into three phases–pre-assessment phase, assessment, and post-assessment. We describe the design and methods adopted for each phase below. The key features are summarized in Box 1.

**Box 1—Key features of the scale-up process**

- Wide stakeholder buy-in
- Engagement of State Government at the highest level
- Creating a local evidence base for planning scale-up including geographic information systems
- Building consensus around how to approach potential health systems barriers
- Developing a costed operational plan for scale-up through an inclusive process
- Planning an evidence-based smart expansion that brought services to the greatest number in the shortest possible time
- Focused implementation through a collaborative roll-out model
- Tracking progress

The FHI 360 Office of International Research Ethics (OIRE) determined that this project does not meet the regulatory definition of research and/or research involving human subjects as defined under the Department of Health and Human Services Code of Federal Regulations [45 CFR part 46.102(d)(f)]. FHI 360 OIRE Project #:899555.

### Pre-assessment phase–ensuring stakeholder buy-in

Recognizing the important role of having a government-led process for sustainability, the governments of these eight states were engaged at the highest political/administrative levels on the need to rapidly scale-up PMTCT services. Advocacy visits were paid to the state governors or in their absence, the deputies or ‘first ladies’ (wife of the Governor who wields significant influence especially on maternal health issues at state level). We prepared advocacy packs which included past achievements of the state government in the area of PMTCT/HIV services as well as data showing the remaining scale/burden of HIV and low coverage of services. The need for a government directive to ensure full scale government-led scale-up was one of the advocacy goals. Once the governors were engaged, they issued directives to the respective health ministries to ensure PMTCT scale-up activities. The scale-up process was designed to be state-led and evidence-driven supported by multiple stakeholders.

To achieve a state-led process, we worked with state health ministry officials to understand the gaps and importance of closing them. Scale-up planning meetings were subsequently held in each state. Attendees included directors general and project managers of state AIDS control agencies, state AIDS program coordinators from the ministries of health, directors of primary health, directors of regulatory bodies for the private health sector, primary health care control boards/agencies, donor coordination departments, departments of economic planning and budgeting, among others. At these meetings, attendees reviewed the objectives of the assessments, health facility lists, geographical maps, distribution of logistics hubs, assessment tool, composition of assessment teams and general logistics details. Detailed implementation plans were drawn up following these meetings. The number of teams, tools and hubs were planned such that assessments were conducted over one week in each state. The assessment tools were designed to collect both quantitative and qualitative data and were accompanied by an assessment guide.

### Assessment phase

While a scale-up plan was available at national level, there were no such comprehensive plans at state and local government area (LGA) levels. Not only were there no guiding documents, there was a dearth of state-level information regarding the status of the health system and understanding of the bottlenecks to expand PMTCT services. In response, the eight states conducted rapid health facility assessments (R-HFA) with PEPFAR technical and financial support. The aim of these assessments was to build evidence at the local level and provide a baseline to plan the rapid scale up of services.

**Fig 1:**
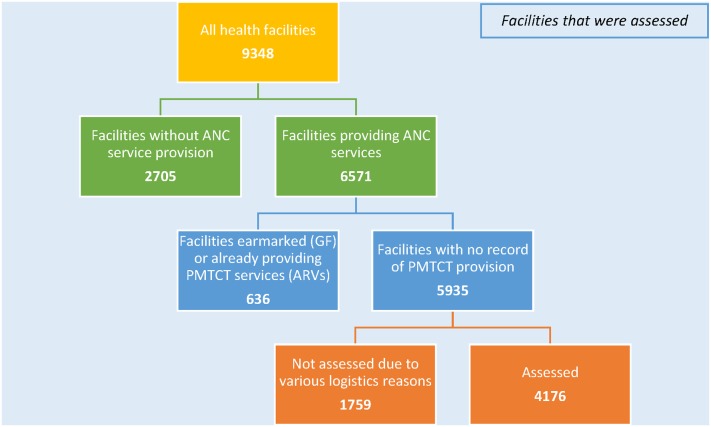
Facilities that were assessed.

The R-HFA was a cross sectional survey using mixed methods conducted in the various states between Feb and May 2013. Data was collected in broad areas categorized as human resource (HR), infrastructure, service availability, utilization of services, enabling environment and community support systems. The components of these broad areas are described in more details below. There were 182 LGAs in the eight 12+1 states. The strengthening integrated delivery of HIV/AIDS services (SIDHAS) project supported HIV services in 150 of these 182 LGAs. The assessment took place in all 150 SIDHAS-supported LGAs in the eight of the 12+1 states. The sampling frame was a total listing of all health facilities in each state as available from different government agencies that kept a form of register of health facilities. The inclusion criterion was all facilities with antenatal care (ANC) services, as in principle these health facilities could provide PMTCT services if equipped with the proper technical and human expertise. Excluded from the assessment were health facilities that were already providing PMTCT services–meaning ARVs for PMTCT—or had concluded plans to initiate PMTCT services in 2013. After applying this criteria, 5935 health facilities were included. Of these, 1759 could not be assessed due to unavailability of comprehensive facility lists in some states, ocean tidal fluctuations, terrain challenges and communal unrest (Fig 1).

Quantitative information across seven domains was collected: facility health linkages (distance and travelling time to facilities offering comprehensive HIV services), health human resource complement (number of staff allocated and available for different cadres), client flow (data showing utilization of services at the health facility including out-patient department, ante-natal clinic and labour ward), scope of services provided (outpatient, inpatient, laboratory, drug dispensing, referrals, etc), community support systems (attached ward development committees, community development associations, etc), current infrastructure (electricity, water, toilets, incinerator, computer, filing cabinets, furniture, medical equipment, drug storage facilities, etc), and future prospects for expansion (availability of space to set up service delivery points or provide additional services). Geospatial location of the facilities was determined using GPS devices. Key informant interviews (KIIs) with health workers were used to explore community birth site options, perceived reasons for preferred choice of birth site, factors influencing facility patronage, and the extent of community participation in service delivery.

Data was collected using Google Forms, exported to MS Excel and analysed using SPSS. The data was then validated with a wide group of state level stakeholders. The human resource complement was measured against the stipulated national standard for a PMTCT site—at least one doctor, at least one nurse midwife, two or more Community health workers (CHEWS/JCHEWS), Pharmacist or Pharmacy technician, Lab scientist and Record officer [12].

### Post- assessment phase

#### Drawing up costed State operational plans to achieve elimination of mother-to-child transmission of HIV (eMTCT) by 2015

From June to September 2013, stakeholders from national, state and local government levels as well as development partners, civil society, traditional institutions and the public converged at state-level workshops to review the R-HFA data and agree on key steps to ensure that 90% of HIV positive pregnant and lactating women have access to ARVs and other PMTCT services. The results of the R-HFA informed areas of focus for each state. In one of the states, Anambra, UNICEF also conducted a bottleneck analysis which focused on identifying the bottlenecks in effective implementation of PMTCT at the local government level; further enriching the gap analysis and planning for specific activities in that state.

In most states there was a discussion around–“with two years to the 2015 deadline, should the operational plans focus on what states think is achievable or should the plans cover what needs to be done to achieve elimination of new pediatric infection”. In seven of eight states, consensus was built to plan for “what needs to be done” and to commit resources to achieving set goals. Consequently, key deliberations were held to modify the national HR and service delivery requirements, with consideration for resources at the state level while not compromising service quality. The minimum state-specific HR complement required per health facility was iteratively refined to determine the number of health workers required to achieve the desired population coverage. The iterative process meant that when a minimum complement was set, it was examined against the possible coverage that will be achieved. If the agreed complement did not achieve a desired coverage, it was refined until a desired coverage level was attained. State-level technical experts then generated a set of key activities required to meet the respective state “elimination” goals as contained in the National PMTCT guidelines [13] and which derived from global eMTCT goals [5]. The activities were relevant to the state context, comprehensive [13] and had strong demand creation components–mobilization, sensitization, media messaging, community level activities, etc. Activities were subsequently refined and costed while an estimation of the impact of implementation to scale was modelled. The operational plans covered the period 2013 to 2015, and had resource commitments from partners at the table including all levels of government—local, state and federal as well as donor agencies. The full list and cost of all the activities are contained in the state level operational plans for eMTCT which are published online [14].

#### Modelling

We constructed a model to estimate the impact of implementation of the operational plans. The objective was to understand (in terms of lives saved, infections averted) if targets were met and plan was implemented to scale. The base case scenario was if current levels of coverage were maintained across three main targets: 1) reduce HIV incidence by 50% among women of reproductive age by 2015; 2) reduce unmet need by 90% of HIV positive women (by increasing voluntary FP use) by 2015; and 3) increase access to antiretroviral treatment (ART) to 90% of HIV positive pregnant women by 2015. The alternate scenario is when these targets are met. The estimated impact is the difference between the two scenarios. The full definitions of variables and assumptions that went into the model are described elsewhere. [6]

#### Smart expansion

With the completion of the costed eMTCT operational plans, implementation began in earnest in October 2013. A key first step in *smart* expansion is the use of available evidence to prioritize areas with the highest MTCT potential (maternal HIV) and widest PMTCT coverage gaps. The LGA was chosen as the intervention unit. To prioritize the facilities, we created a rank order matrix applied at two levels–to prioritize LGAs and within LGAs, to prioritize facilities. The rank order was designed to select LGAs with the highest PMTCT coverage gaps, high HIV prevalence and large population size. The HIV prevalence, estimated population of women of reproductive age group and fertility rate was used to derive an estimated number of HIV positive pregnant women for each LGA. This represented the burden of potential MTCT in each LGA. A rank (rank 1) was assigned to each LGA for this burden–the higher the burden, the higher the rank. A second rank (rank 2) was assigned for PMTCT service coverage gap. Using data from the assessment, we calculated the proportion of sites with ANC services that did not provide PMTCT services. The higher this proportion, the higher the rank 2 value. In essence, rank 2 was assigned to give higher rank to LGAs with wider service coverage gaps. We then summed up both ranks 1 & 2 to give a final rank sum that was used to determine which LGAs were prioritized for the earlier phases of scale-up. An example of the prioritization in Abia State is shown in Table 1.

**Table 1:** Rank order prioritization of LGAs for PMTCT expansion–example from Abia state.

LGAs	MTCT Burden		PMTCT Service Coverage Gap			Rank sum [Rank 1 +Rank 2]
	Estimated number of HIV+ pregnant women	Rank 1 (maternal HIV burden)	Number of sites with ANC services	Proportion without PMTCT services	Rank 2 (PMTCT service gap)	
Umuahia South	995	15	37	100%	17	32
Ikwuano	600	8	39	100%	17	25
Obingwa	792	12	51	98%	12	24
Aba South	1785	17	64	88%	6	23
Arochukwu	737	11	49	98%	12	23
Osisioma	961	14	65	97%	9	23
Umuahia North	1597	16	64	89%	7	23
Bende	839	13	62	95%	8	21
Ugwunagbo	372	3	52	100%	17	20
Ukwa East	253	2	20	100%	17	19
Isiala Ngwa South	595	7	39	97%	9	16
Aba North	446	5	60	97%	9	14
Nneochi	710	10	37	84%	3	13
Ohafia	690	9	44	82%	2	11
Isuikwuato	504	6	44	84%	3	9
Isiala Ngwa North	119	1	44	86%	5	6
Ukwa West	380	4	19	68%	1	5
Total	12,377		790	93%		

Within LGAs, R-HFA data was used to rank the facilities by the number of clinical health workers available, the number of antenatal attendees at the health facility in the 12 months preceding the assessment and the number of deliveries. The higher the numbers reported for these three elements, the more likelihood of being selected for the earlier phase of scale-up. In essence, the rank order prioritization was aimed at selecting facilities that had more HR, were patronized by more pregnant women, had no other facility providing PMTCT in the vicinity and were located in a higher HIV burden LGA. Services were then scaled-up in a phased approach. The equitable spread was mapped using geographic information system data from the R-HFA.

#### Roll-out model

Once a clearer picture for service expansion was painted, the next stage was to roll out services. First a list of health workers to be trained was generated for each selected facility. This was done by the different government agencies overseeing the various levels of health workers in the health system. Government agencies regulating the private-for-profit health sector generated the private health facility training lists in conjunction with proprietors.

Trainings were followed by “site activation”. Activation in the scale-up context involved a two-day post-training onsite hands-on support to respective facilities. Activation teams spent this time deploying activation kits, setting up PMTCT services and getting health workers acquainted with service flow. The activation kits included HIV rapid test kits, ARVs, patient education and information materials, data collection and reporting tools. Further, mobilization and sensitization activities were conducted in communities around the health facilities with the aim of generating demand for these services.

**Fig 2:**
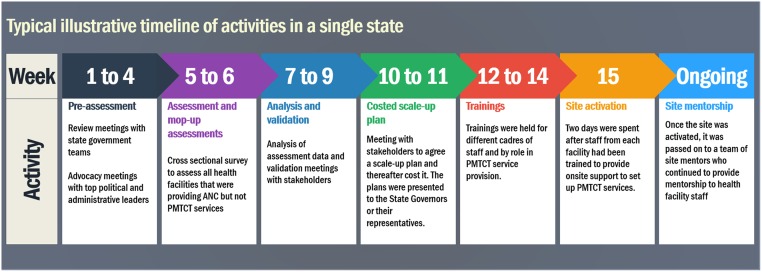
Typical timeline for activities within a single state.

The training and activation phase for each facility was designed to be led by multi-disciplinary teams and concluded within a month. This meant that the team of health workers from each facility went through requisite training courses and their facility was visited for the two-day site activation all within one month. Resources were deployed accordingly to work within this timeline. Thus, concurrent training and site activation batches were set up in a production line fashion. As sites were trained by the multiple training teams at different training venues, they were passed on to activation teams who provided onsite mentorship for commencement of PMTCT services as well as supply of essential commodities. The typical timeline is depicted in Fig 2 below.

Fig 2 shows the typical timeline of activities within a single state. Activities could however stretch into longer periods especially for such activities that required securing appointments or scheduling with political office holders. Activities were not being run simultaneously in all 8 states, therefore, activities in ‘week 1’ could in real dates, when aggregated, have spanned a total of eight to 12 weeks across the eight different states.

Once training and activation were completed, the new PMTCT sites were handed over to site mentors. A quality mentorship and accountability program (Q-MAP) was instituted to provide routine mentorship of health care workers in these newly activated sites. These site mentors comprised of experienced multidisciplinary PMTCT technical experts (clinicians, pharmacists, laboratory scientists, monitoring & evaluation officers) from the supporting NGO and Government staff. They paid routine visits–at least fortnightly in the first two months and monthly thereafter–to review guidelines, standard operating procedures, client folders, and service delivery challenges.

A unique feature of the roll-out phase was the inclusion of the private and non-formal health sector. The expansion incorporated specific strategies to engage privately owned health facilities while the non-formal health sector included traditional birth attendants and other community leaders. Further emphasis was made on strong demand creation given the low rates of health facility utilization for maternal and child health services.

### Ethics statement

This paper reports data from health system processes. The subjects are not directly human but the health system processes that led to increased access for the population. Informed consent was not obtained as no clinical records were retrieved for this paper. Only routine health system data–service statistics–are presented. The routine data has no identifiers and is not linked to individual persons. The FHI 360 Office of International Research Ethics (OIRE) has determined this project does not meet the regulatory definition of research and/or research involving human subjects as defined under the Department of Health and Human Services Code of Federal Regulations [45 CFR part 46.102(d)(f)]. Given this determination, further review and approval of this project is not required. FHI 360 OIRE Project #:899555.

## Results

### Galvanized health system

The wide engagement of stakeholders and communities created a conducive environment for expansion of PMTCT services. Participants at the different meetings became key champions for the scale-up in their constituencies. The state governments showed early commitment to the operational plans developed by funding a local level dissemination to the state governors in five of the eight states. Funds were immediately earmarked to operationalize the plans.

The comprehensive process adopted, including multi-sectoral stakeholder participation, helped mitigate some health system constraints quite quickly in some states. Being part of the process gave senior government officials and some private sector owners a firsthand understanding of the urgent need to address the gaps in PMTCT service delivery. In some states, government engaged or redistributed staff. In others, commitments were made to engage more staff and upgrade some facilities. In many states, facilities that had junior cadre health workers were clustered and assigned to physicians and pharmacists for regular support. In all eight states, a total 7224 health workers were trained for PMTCT service provision. In one state, the executive governor funded all rapid test kits that will be needed as a direct intervention of the state government. Some private sector owners immediately committed to making free testing of pregnant women available in their facilities. These commitments helped to overcome some critical challenges that may have limited the scope of the scale-up.

### Key assessment findings

**Fig 3:**
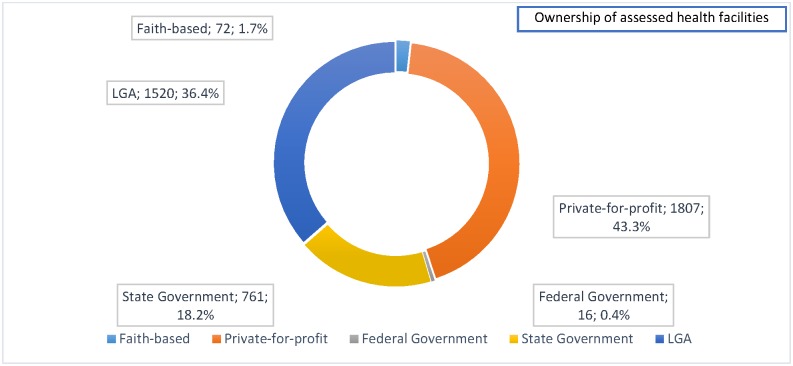
Ownership of assessed health facilities.

The assessment highlighted a number of key findings. Firstly, more than four of every 10 facilities without PMTCT services were private-for-profit facilities (Fig 3).

Fig 3 shows that private-for-profit facilities constituted 43.3% of facilities assessed.

**Fig 4:**
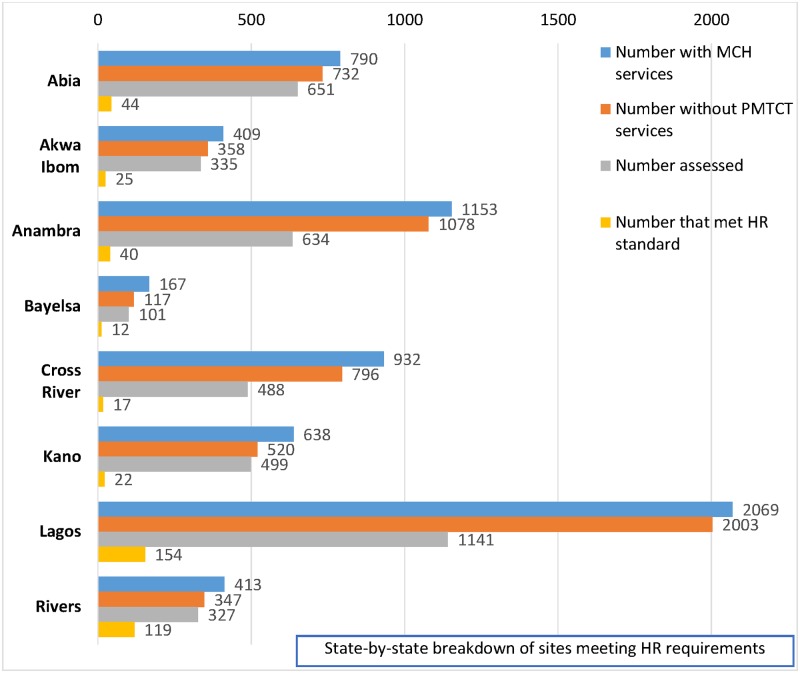
State-by-state breakdown of sites meeting HR requirements.

Secondly, at an aggregate level, only 10.4% (433) of all assessed 4176 facilities met stipulated national human resource standards for PMTCT service provision [12]. Of the eight states, the state with the highest proportion of assessed facilities meeting national standards, had only 36% of assessed facilities meeting the national criteria (Fig 4).

Only a small proportion of assessed facilities met stipulated human resource requirements for PMTCT service provision. Lagos state had the highest absolute number of facilities meeting the HR standard (154) but Rivers state had the highest proportion of assessed sites meeting the HR standards.

Thirdly, availability of services into which PMTCT could be integrated varied for different items but overall, more than half of assessed facilities had either the requisite service or infrastructure. Close to nine of every 10 assessed facility provided routine drugs for antenatal clients. Maternity services were available in 74% of facilities assessed. For most services except for child follow-up services, family planning, and space for confidential counseling more private facilities than public had the services (Table 2).

**Table 2:** Availability of services and infrastructure for PMTCT scale-up.

Infrastructure and service readiness	Facilities assessed		
	Public	Private	Total
	2297	1879	4176
Dispensing haematenics and IPTPs	88%	76%	83%
Antenatal care space	85%	74%	80%
Provides maternity services	71%	78%	74%
Provides child follow-up services	82%	64%	74%
Has space for labour and delivery	64%	78%	70%
Provides family planning services	70%	61%	66%
Has a pharmacy store/dispensary	58%	65%	61%
Available laboratory services	55%	67%	60%
Has a medical records system	46%	61%	53%
Has designated space for laboratory	46%	59%	52%
Has space for confidential counselling	61%	38%	51%

Fourthly, facilities providing PMTCT services were mostly clustered around urban centres. Fifthly, most private sector facilities were not linked with supporting community systems (e.g. ward development committees) when compared to their public counterparts. Finally, qualitative data showed that in health workers’ opinion, improved infrastructure, a bolstered workforce, and enhanced working conditions were necessary to achieve improved utilization of health facilities by communities. The full results of the assessments are available online. [6]

### Improvement in PMTCT coverage

**Fig 5:**
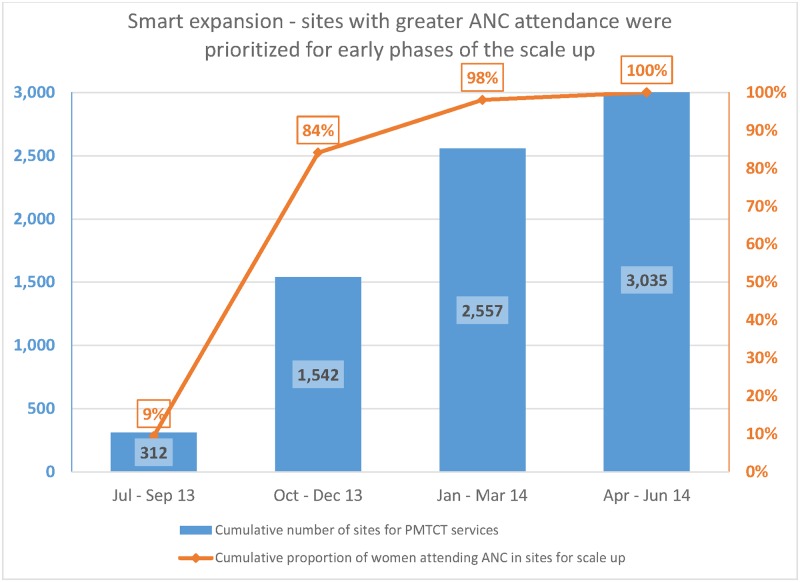
Smart expansion—sites with greater ANC attendance were prioritized for early phases of the scale up.

With the adoption of smart scale-up, our phased approach put 51% (1542 facilities) of the planned scale-up in the first two quarters (July 2013 to Dec. 2013). Using ANC records, these 1542 facilities are those patronized by 84% of the women expected to attend ANC in all facilities planned for scale-up (Fig 5).

Fig 5 depicts the smart scale-up approach which selected facilities with the greatest need for the earlier phases of scale-up. About 84% of the pregnant women who attended ANC in facilities targeted for scale-up were reached in the 1230 facilities activated for services in the quarter from October to December 2013.

A total of 2044 facilities were eventually supported to begin provision of PMTCT services over a period of 10 months between June 2013 and March 2014. The bulk of the 2044 facilities (1969, 96.3%) were activated between September 2013 and March 2014. This moved facility coverage with PMTCT services from 8% to 50% with varying results across the eight states (Table 3).

**Table 3:** Increase in facility PMTCT coverage from 8% to 50% in eight states.

State	No of facilities with ANC	Number of facilities with PMTCT (ARVs) at R-HFA	% facility coverage prior to scale up	Facilities activated as at March 2014	Global Fund scale-up	Total number of facilities providing PMTCT services as at Mar 2014	% facility coverage as of March 14 (facilities providing PMTCT as a proportion of facilities with ANC)
**Abia**	790	46	6%	386	12	444	56%
**Akwa Ibom**	409	34	8%	301	12	347	85%
**Anambra**	1153	51	4%	293	12	356	31%
**Bayelsa**	167	38	23%	86	12	136	81%
**Cross River**	932	134	14%	380	12	526	56%
Kano*	273	34	12%	233	0	267	98%
Lagos*	934	10	1%	120	12	142	15%
**Rivers**	413	65	16%	245	12	322	78%
**Total**	5052	412	8%	2044	84	2540	50%

* Data for Lagos and Kano States are from the 10 of 20 and 22 of 44 LGAs supported by SIDHAS respectively

A 246% increase has also been recorded in the number of pregnant women and their families who now have access to HIV testing and counselling in the context of PMTCT. Similarly, access to antiretrovirals for PMTCT has witnessed a 152% increase in these eight states between October 2013 and October 2014 (Table 4).

**Table 4:** Increased population level access to PMTCT.

	Testing in PMTCT					ARVs for PMTCT				
	Oct-12 to Sep-13		Oct-13 to Sep-14		Change	Oct-12 to Sep-13		Oct-13 to Sep-14		Change
	Estimated no. of pregnant women	Pregnant women counselled, tested and received results (CTRR)	Estimated no. of pregnant women	Pregnant Women CTRR	Percentage increase in number of pregnant women tested	Estimated number of HIV+ pregnant women	HIV+ Pregnant Women receiving ARVs for PMTCT	Estimated number of HIV+ pregnant women	HIV+ Pregnant Women receiving ARVs for PMTCT	Percentage increase in number of HIV+ pregnant women receiving ARVs
Abia	173,031	17,859	177,766	82,185	360%	12,631	722	12,977	1,663	130%
Akwa Ibom	249,641	25,431	258,275	144,373	468%	27,211	1,276	28,152	4,248	233%
Anambra	252,261	57,080	257,836	163,788	187%	21,947	1,315	22,432	2,548	94%
Bayelsa	105,144	46,812	108,215	73,075	56%	9,568	449	9,848	585	30%
CRS	177,777	37,935	182,791	155,354	310%	12,622	1,051	12,978	3,080	193%
Kano	579,121	84,232	617,155	270,990	222%	19,690	517	20,983	1,031	99%
Lagos	1,094,152	38,627	1,129,165	122,662	218%	55,802	1,079	57,587	1,565	45%
Rivers	332,442	24,908	343,779	139,596	460%	19,947	527	20,627	2,734	419%
Total	2,963,569	332,884	3,074,982	1,152,023	246%	179,417	6,936	185,584	17,454	152%

Not only were facilities (public and private) activated, communities were engaged and traditional birth attendants were also linked to facilities for a continuum of care for pregnant women.

The modelling exercise showed that if the scale-up plans were implemented in their entirety, a combined 6,824,063 disability adjusted life years—DALYs will be saved in the eight states while 127,870 infections among HIV-exposed babies will be prevented. This will be at a combined cost of 96 billion naira (about 640 million USD). The state level details have been published online. [6]

## Discussion

The process used to rapidly scale–up PMTCT services in eight of the 12+1 highest burden states of Nigeria was rapid, yet thoughtful and highly participatory. Reflections on this experience highlights a number of findings and lessons.

In many countries, issues with the health system are longstanding, limit PMTCT scale-up and do not appear would be resolved before the 2015 eMTCT target. [15] Same can be said of Nigeria. Countries that have made progress have however worked around these health system challenges [16] including working with communities. [17] This point is important from our perspective–scale-up could only have been possible because there was a consensus to work around health system challenges.

To begin, building consensus for scale-up had far reaching effects. Multiple stakeholder buy-in has been described as an essential step for successful scale up of services towards eMTCT. [16] Indeed this could have accounted for the achievements throughout the entire scale-up described in this paper. A deliberate attempt was made at the outset and this paid significant dividends down the line.

We also discovered that local data is considerably more powerful to local stakeholders than national data. The national PMTCT scale-up plan 2010–2015 which proposed decentralization to lower levels of health care as a key strategy for closing the coverage gaps [18] has remained a national level document with limited operationalization at sub-national levels. The disparate magnitude of mother-to-child-transmission within and between states was only obvious when stakeholders were presented with data. In fact, a governor from one of the states remarked, “do we still have a burden this large…so what have we been doing all this while?” This lesson is consistent with other efforts to scale-up PMTCT services. For example, a local review of data with primary stakeholders was one of the key steps in a South African initiative to attain eMTCT. [16] National requirements for PMTCT service provision may also have served as a barrier to scale up. With only 10% of health facilities meeting these requirements, scale-up strictly along these requirements would have been severely restricted.

Adopting *smart* expansion brought services first to those who needed it most. Data guided scale-up which prioritized the commencement of services, ensuring that services were first taken to facilities where they would achieve the greatest population level impact. Starting scale-up from areas with higher HIV prevalence makes it more likely to achieve higher PMTCT uptake as found by Audureau et al. [19] *Smart* expansion has recently become a focus of program managers gaining sufficient coverage at the 2014 International AIDS Society conference held in Australia.

Next, our intervention may corroborate the established fact that community-based activities must complement facility-based service provision. Improvements in facility coverage alone however is not sufficient to reach elimination. [20] Uptake of services provided at facilities can be significantly improved through dedicated demand creation activities in communities. [21] The Government of Nigeria has set forth demand creation strategies [8] which were informed by locally generated evidence. It is expected that these will complement increased facility coverage to achieve a wider population coverage and uptake of services.

Furthermore, a key R-HFA finding was the critical role of the private health sector in increasing coverage of PMTCT services. Before scale-up, available PMTCT services were largely concentrated in public sector facilities which provided services for only a fraction of the population. It is estimated that more than 60% of Nigerians receive health services through the private sector, few of which offer PMTCT services. [7] Early results reinforce the importance of the private health sector; in 2014, private health facilities constituted 25% of SIDHAS-supported PMTCT sites and have contributed 23% of pregnant women counselled and tested for HIV. Being relatively newcomers to the PMTCT program, targeted efforts to bring the private sector on-board were necessary.

Finally, it is important to highlight the fact that while challenges were encountered, they were not insurmountable. The literature is awash with barriers to scale-up, limitations to implementation, and similar issues [2, 15, 19, 22–24]. Encouraging, however, is the fact that many of these barriers are manageable. Dedicated and committed leadership can marshal resources and galvanize action from various stakeholders in order to address the barriers and move forward [21].

For example, it has been argued that while funding shortages may prove a difficult barrier for PMTCT scale-up in developing countries, the more dire challenge is that of human resource shortage. [25] The R-HFA results were consistent with this argument; over 50% of assessed facilities had the requisite infrastructure or service platform, while only 10% had requisite HR complement. Smart scale-up, agreed human resource criteria, involvement of key stakeholders, deployment of staff, clustering of technical support to junior cadre health workers, and in-service trainings were some of the strategies that helped address human resource shortfalls. Nevertheless, some facilities could not be included in the scale-up because of severe human resource shortages.

## Conclusion

Some countries in sub-Saharan Africa are struggling to achieve their desired PMTCT coverage levels. This report demonstrates that a data-driven and participatory approach can be used to rapidly scale-up PMTCT services at community and facility levels in this region. Crucial to the scale-up process in Nigeria was the collection and dissemination of local data, which made possible an accurate understanding of the strengths and weaknesses of the health system and allowed for an informed prioritization of facilities in the scale-up plans. Another critical ingredient is the participation and commitment of a wide group of stakeholders across all levels of the health system. Effective stakeholder involvement contributes to the implementation of innovative strategies to address bottlenecks, such as human resource constraints, and enables additional financial resources to be earmarked for PMTCT services. These results present us with hope for real progress in Nigeria. We are confident that the efforts described here will contribute significantly to eliminating new pediatric HIV infection in Nigeria.

## Supporting Information

S1 File Assessed Facilities. This file contains data on the type of facilities that were assessed across eight states as displayed in Fig 1.
(XLSX)

S2 File Ownership. Pulled from R-FHA reports, the underlying data that describes facilities that were assessed by the ownership of the facilities as shown in Fig 3.
(XLSX)

S3 File Standards. In this file, data about facilities that met the human resource standards is presented. The data is summarized in Fig 4.
(XLSX)

S4 File Services and infrastructure availability. S4 File makes available, data summarized in Table 2 about availability of services and infrastructure that can serve as a platform for PMTCT services.
(XLSX)

S5 File Smart expansion_greatest need first. Facilities with greater utilization were prioritized for earlier phases of scale-up. This file presents the data in this regard as shown in Fig 5.
(XLSX)

S6 File Improvements in PMTCT coverage. Data on coverage of services is presented in S6 File.
(XLSX)

## Abbreviations

ANC: antenatal care
ARVs: antiretrovirals
eMTCT: elimination of mother-to-child transmission of HIV
HIV: human immunodeficiency virus
HTC: HIV testing and counseling
LGA: local government area
PLHIV: persons living with HIV
PMTCT: prevention of mother-to-child transmission of HIV
R-HFA: rapid health facility assessment
SIDHAS: strengthening integrated delivery of HIV/AIDS services
